# Draft Genome Sequence of Enterococcus durans OSY-EGY, a Multiple-Antimicrobial-Peptide Producer Isolated from Egyptian Hard Cheese

**DOI:** 10.1128/MRA.00303-19

**Published:** 2019-07-11

**Authors:** Walaa E. Hussein, Lingzi Xiaoli, Ahmed E. Yousef

**Affiliations:** a Department of Food Science and Technology, The Ohio State University, Columbus, Ohio, USA; b Department of Food Science, The Pennsylvania State University, University Park, Pennsylvania, USA; c Department of Microbiology, The Ohio State University, Columbus, Ohio, USA; University of Delaware

## Abstract

Enterococcus durans OSY-EGY was isolated recently from cheese. The strain produces potent antimicrobial agents. Here, we present the draft genome sequence of the strain, with a genome size of 3,230,625 bp and an average G+C content of 37.69%. Draft genome mining identified several biosynthetic gene clusters encoding multiple antimicrobial peptides.

## ANNOUNCEMENT

Enterococci are lactic acid bacteria found in the gastrointestinal tract and are encountered as natural microbiota in fermented food (1–3). Some enterococci produce antimicrobial peptides that are useful as protective agents against foodborne pathogens (4). Enterococcus durans OSY-EGY was isolated from imported Egyptian cheese, after sample homogenization, dilution, and spread plating onto de Man-Rogosa-Sharpe (MRS) agar medium (Oxoid, Thermo Scientific, Waltham, MA, USA), and the plates were incubated at 30°C for 72 h. Hundreds of colonies were screened for antimicrobial activity, as described previously (5). OSY-EGY was found to inhibit Listeria monocytogenes, Staphylococcus aureus, Enterococcus faecalis, and Bacillus cereus. To reveal the genetic basis of the antimicrobial activity of *E. durans* OSY-EGY, the strain’s whole genome was sequenced.

A single colony of OSY-EGY was cultivated in MRS broth for 24 h at 30°C. The genomic DNA (gDNA) was extracted and purified using the DNeasy blood and tissue kit (catalog number 69504; Qiagen, Valencia, CA), and its concentration was determined spectrophotometrically (ND-1000 spectrophotometer; Thermo Fisher Scientific). The gDNA was used to construct the paired-end library using the Nextera XT DNA library preparation kit (catalog number FC-131-1024; Illumina), as described by the kit manufacturer. The constructed library fragment sizes were evaluated using a Bioanalyzer 2100 (Agilent Technologies, Inc., Coralville, IA) and the Agilent high-sensitivity DNA kit (catalog number 5067-4626). The library’s DNA concentration was measured spectrophotometrically. Illumina MiSeq next-generation sequencing was performed using the MiSeq reagent kit v2 for 500 cycles (catalog number MS-102-2003) to generate 2 × 250-bp paired-end reads. Raw read quality (score of 36) was determined using FastQC 0.11.7 (https://www.bioinformatics.babraham.ac.uk/projects/fastqc/), followed by *de novo* assembly using SPAdes 3.10.1 (6), with default parameters. The assembly generated 227 contigs (maximum contig size, 179,356 bp; minimum contig size, 203 bp), a genome size of 3,230,625 bp, an *N*_50_ value of 52,003 bp, and an average G+C content of 37.69%, calculated by Artemis software (7), with default parameters. Contig ordering was done against a reference genome, that of *E. durans* strain KLDS 6.0933, using Mauve 2.4.0 (8). The average nucleotide identity (ANI) values between the genome of OSY-EGY and three sequenced *E. durans* genomes were calculated using the JSpeciesWS Web server (9). The results indicated that OSY-EGY shared the highest (99.67%) identity with *E. durans* KLDS6.0933, followed by *E. durans* KLDS6.0930 (99.64%) and *E. durans* BDGP3 (97.82%). Draft genome annotation was performed using the NCBI Prokaryotic Genome Annotation Pipeline (10), which revealed 3,029 coding sequences, 64 tRNAs, and 14 rRNA genes.

Genome mining using BAGEL 3.0 (11) and antiSMASH version 4.0.3 (12) revealed that OSY-EGY genetically encoded two novel lantibiotics, one novel enterocin, and two enterocins previously reported as enterocin L50A and enterocin L50B. In conclusion, *E. durans* OSY-EGY draft genome sequencing gave a better understanding of the genetic basis of the strain’s antimicrobial characteristics.

### Data availability.

The sequence raw reads and assembled genome of Enterococcus durans OSY-EGY were deposited in NCBI under BioProject accession number PRJNA517524. The raw reads have been deposited at the Sequence Read Archive (SRA) under the accession number SRR8506815. The assembled genome has been deposited at DDBJ/ENA/GenBank under the accession number SEHG00000000. The version described in this paper is version SEHG01000000.
